# Indexation of left ventricular mass to predict adverse clinical outcomes in pre-dialysis patients with chronic kidney disease: KoreaN cohort study of the outcome in patients with chronic kidney disease

**DOI:** 10.1371/journal.pone.0233310

**Published:** 2020-05-19

**Authors:** Sung Woo Lee, Hyang Ki Min, Dong-Wan Chae, Kook-Hwan Oh, Curie Ahn, Wookyung Chung, Joongyub Lee, Yong-Soo Kim, Su Ah Sung, Representing the KNOW-CKD Study Group

**Affiliations:** 1 Department of Internal Medicine, Nowon Eulji Medical Center, Eulji University, Seoul, Republic of Korea; 2 Department of Internal Medicine, Seoul National University Bundang Hospital, Seongnam, Republic of Korea; 3 Department of Internal Medicine, Seoul National University College of Medicine, Seoul, Republic of Korea; 4 Department of Internal Medicine, Gachon University Gil Medical Center, Incheon, Republic of Korea; 5 Department of Prevention and Management, Inha University Hospital, Incheon, Republic of Korea; 6 Department of Internal Medicine, The Catholic University of Korea, Seoul St. Mary's Hospital, Seoul, Republic of Korea; Kaohsiung Medical University Hospital, TAIWAN

## Abstract

**Background:**

No study has compared the clinical impact of indexation of left ventricular mass (LVM) on adverse clinical outcomes in pre-dialysis patients with chronic kidney disease (CKD).

**Methods:**

We reviewed 2,101 patients from a large-scale multi-center prospective study that gathered anthropometric and echocardiographic measurements and clinical outcomes. The LVM was indexed as body surface area (LVMI-BSA) and height raised to the power of 2.7 (LVMI-H2.7). The main outcomes were composite renal and cardiovascular events and all-cause mortality. Left ventricular hypertrophy (LVH) was defined as the highest sex-specific quartile of LVMI-BSA or LVMI-H2.7.

**Results:**

During a mean period of 3.5 years, 692 patients developed composite outcomes (32.9%). The area under the curve at 5 year of LVM (60.6%) for composite outcome was smaller than that for LVMI-BSA (63.2%, *P* <0.001) and LVMI-H2.7 (63.4%, *P* <0.001). The hazard ratio (HR) and 95% confidence interval (CI) per one unit increase in LVM (g), LVMI-BSA (g/m^2^), and LVMI-H2.7 (g/m^2.7^) for composite outcomes were 1.004 (1.002–1.005, *P* <0.001), 1.011 (1.006–1.016, *P* <0.001), and 1.023 (1.012–1.035, *P* <0.001), respectively. Patients with LVH determined by LVMI-BSA and LVMI-H2.7 (HR 1.352, 95% CI 1.123–1.626, *P* = 0.001) and LVH determined by only LVMI-BSA (HR 1.908, 95% CI 1.233–2.953, *P* = 0.004) showed an independent increase in the risk of composite-outcome development, when compared with patients without LVH, according to LVMI-BSA and LVMI-H2.7.

**Conclusion:**

Indexation of LVM improved the prediction of adverse outcomes. BSA may be as useful as height^2.7^ in indexing of LVM for predicting adverse outcomes in pre-dialysis patients with CKD.

## Introduction

Left ventricular (LV) mass (LVM) increases in response to pathophysiological stresses, resulting in LV hypertrophy (LVH) [1]. Pressure overload (i.e., hypertension) causing concentric LVH and volume overload (i.e., valvular disease) causing eccentric LVH are two major forms of stress [1]. LVH is associated with an increased risk of cardiovascular events and mortality [2–4], and regression of LVH is associated with a reduction in cardiovascular morbidity and mortality [2]. Patients with chronic kidney disease (CKD) are at higher risk of cardiovascular events [5]. LVH is a common problem in these patients [6], causing significant morbidity and mortality [7–9].

The size of a normal heart is influenced by sex, exercise, age, and ethnicity (1). LVM is influenced by body size. Appropriate indexing of LVM is necessary to minimize over- or under-estimation of LVH (1). Body surface area (BSA) and height raised to the power of 2.7 power (height^2.7^) are common indexing parameters [10–13]. Although the American Society of Echocardiography (ASE) guideline [1, 14] has defined LVH using LVM indexed with BSA (g/m^2^), indexing LVM (LVMI) with BSA in patients with CKD is questionable, because body-fluid volume status is unstable in such patients [15]. Height^2.7^ has been recommended as a more appropriate method for indexation in patients with CKD than that using BSA, because Zoccali et al. reported better prognostic impact of LVMI-H2.7 than LVMI-BSA in patients undergoing dialysis [16]. Nevertheless, the results from patients with CKD undergoing dialysis cannot be directly applied to patients with CKD in pre-dialysis, because clinical conditions vary according to the stage of CKD [17]. Therefore, we identified the best indexation for LVM for predicting adverse clinical outcomes in patients with CKD in pre-dialysis using a large number of adults enrolled in the KoreaN cohort study for Outcome in patients With Chronic Kidney Disease (KNOW-CKD).

## Material and methods

### Participants

The KNOW-CKD was a multi-center prospective cohort study that included 2,238 patients with CKD stages 1–5 who were in pre-dialysis, enrolled between 2011 and 2016 in Korea. The detailed design and methods of the KNOW-CKD have been published earlier (NCT01630486 at http://www.clinicaltrials.gov) [18]. The estimated glomerular filtration rate (eGFR) was calculated using the Chronic Kidney Disease Epidemiology collaboration creatinine equation [19]. CKD and its stages were defined using the Kidney Disease Improving Global Outcomes 2012 guidelines [20].

We excluded 137 patients from the cohort of 2,238 participants because of missing echocardiographic measures, anthropometric measures, and clinical outcomes in 101, 14, and 22 patients, respectively. Finally, 2,101 patients were included.

### Ethics statement

The protocol of the KNOW-CKD adhered to the principles of the Declaration of Helsinki and was approved by the Institutional Review Boards (IRB) of each participating hospital, including Seoul National University Hospital (IRB number: 1104-089-359), Yonsei University Severance Hospital (IRB number: 4-2011-0163), Kangbuk Samsung Medical Center (IRB number: 2011-01-076), Seoul St. Mary’s Hospital (IRB number: KC11OIMI0441), Gil Hospital (IRB number: GIRBA2553), Nowon Eulji Medical Center (IRB number: 201105–01), Chonnam National University Hospital (IRB number: CNUH-2011-092), and Busan Paik Hospital (IRB number: 11–091). Written informed consent was obtained from all participants. All data were fully anonymized before we accessed them.

### Measurement of left ventricular mass index

Complete two-dimensional M-mode echocardiography and Doppler studies were performed in the standard manner by cardiologists of the participating hospitals, who were blind to the clinical data. M-mode examination was performed according to the American Society of Echocardiography guidelines [14]. Left ventricular end diastolic diameter (LVEDD), left ventricular end systolic diameter (LVESD), inter-ventricular septum thickness (IVST), left ventricular posterior wall thickness (LVPWT), left atrial diameter (LAD), regional wall motion abnormality (RWMA) and ejection fraction (EF) were recorded with echocardiography. LVM was determined using the Devereux formula [14]: LVM (g) = 0.8 × {1.04 × [(LVEDD + IVST + LVPWT)^3^ - (LVEDD)^3^]} + 0.6. LVMI was calculated by normalizing LVM with height^2.7^ (LVMI-H2.7, g/m^2.7^) or BSA (LVMI-BSA, g/m^2^). BSA was calculated using the Du Bois formula: BSA (m^2^) = 0.007184 × (height in cm) ^0.725^ × (weight in kg)^0.425^ [21]. The first, second, third, and fourth quartiles for LVM were 59.0–128.0 g, 128.0–155.9 g, 155.9–186.4 g, and ≥186.4 g, respectively.

### Definition of LVH

LVH was defined as the highest sex-specific quartile of LVMI in the study’s patients: ≥48.6 g/m^2.7^ in men and ≥48.4 g/m^2.7^ in women defined by LVMI-H2.7 (LVH-H2.7) and ≥109.9 g/m^2^ in men and ≥100.5 g/m^2^ in women defined by LVMI-BSA (LVH-BSA). We classified the patients into four combined LVH groups, according to the two LVMIs: no LVH/both group (no LVH with LVMI-H2.7 or LVMI-BSA), LVH/BSA-only group (LVH determined only with LVMI-BSA), LVH/H2.7-only group (LVH determined only with LVMI-H2.7), and LVH/both group (LVH determined with both, LVMI-BSA and LVMI-H2.7).

### Definitions of study outcomes

Composite renal and cardiovascular events, and all-cause mortality were the primary outcomes. Outcome measurements were described in detail by previously published protocol [18]. A renal event was defined by a >50% decrease in eGFR from the baseline values, doubling of serum creatinine, or initiation of dialysis or kidney transplantation. A cardiovascular event was defined as any first event of the following since study enrollment: acute myocardial infarction, unstable angina, percutaneous coronary artery intervention or coronary bypass graft surgery, ischemic or hemorrhagic cerebral stroke, congestive heart failure and other major cardiovascular events that required hospitalization, interventions, or therapy during the follow-up. Patients with CKD stage ≥ 3 were under close observation and had been followed up at 1- to 3-month intervals by all participating centers. Patients who reached the endpoints were reported by each center, irrespective of the study protocol. Patients were followed up till December 31, 2018 or until they dropped out or died. All the adverse outcomes were detected and adjudicated annually by the researchers and adjudication committee [18].

### Other measurements and definitions

Clinical data, including detailed demographic information and baseline laboratory results, were extracted from the electronic data management system (PhactaX). Hypertension was defined as systolic blood pressure (BP) ≥140 mm Hg or diastolic BP ≥90 mmHg or treatment with anti-hypertensive drugs. Diabetes was defined as fasting plasma glucose ≥126 mg/dL, or treatment with insulin or oral anti-diabetic drugs. Body mass index (BMI) was calculated as weight (kg) per square meter of height (m^2^).

### Statistical analysis

The distributions of continuous variables were evaluated using histograms and Q-Q plots. Two variables, high sensitivity C-reactive protein (hsCRP) and urine protein-to-creatinine ratio (UPCR) were not normally distributed. Normally distributed continuous variables, non-normally distributed continuous variables, and categorical variables were expressed as mean ± standard deviation, median (interquartile range), and percentages, respectively. The *P*-trend was analyzed with a linear-term of one-way analysis of variance (ANOVA for normally distributed continuous variables), with the Jonckheere-Terpstra test for non-normally distributed continuous variables, and with linear-by-linear association for categorical variables. Differences were analyzed using Bonferroni post-hoc analysis of one-way ANOVA for normally distributed continuous variables, Mann–Whitney U tests for non-normally distributed continuous variables, and chi-squared tests for categorical variables. The hazard ratio (HR) and its 95% confidence interval (CI) of LVM and its indexations for study outcomes were assessed using Cox proportional hazard regression analysis. The assumption of proportional hazard was tested using the log minus log plot for categorical variables and interaction analysis with time covariate using time-dependent Cox regression analysis for continuous variables. When the proportional hazard assumption was not met, time-dependent Cox regression analysis was used for primary exposures (LVM, LVMI-BSA, and LVMI-H2.7), while categorization by median values was done for other covariates: systolic BP (median 127 mmHg) and diastolic BP (median 77 mm Hg), cholesterol (median 4.4 mmol/l), eGFR (median 46.3 ml/min/1.73m2), blood urea nitrogen (median 8.6 mmol/l), bilirubin (median 10.3 μmol/l), albumin (median 42 g/l), and haemoglobin (median 12.8 g/dl). A *P*-value < 0.05 was considered statistically significant. Covariates were chosen based on clinical and statistical relevance for multivariate analysis and only patients without missing values were included in the analysis. The area under the curve (AUC) with the CI of the time-dependent receiver operating characteristic curve (ROC) using Kaplan-Meier estimator was evaluated using R Version 3.6.2 (R Core Team, 2019, R Foundation for Statistical Computing, Vienna, Austria) with “timeROC” package [22]. All analyses (unless specified otherwise) were performed using SPSS Version 22 (IBM Corp. released 2013, Armonk, NY).

## Results

Of the 2,101 patients, the mean age was 53.6 years and 61.0% were men. The causes of CKD were diabetic nephropathy in 24.8%, hypertensive nephropathy in 19.8%, glomerulonephritis in 31.7%, and others in 23.7% of patients. The mean LVM, LVMI-BSA, and LVMI-H2.7 were 161.6 g, 93.2 g/m^2^, and 42.1 g/m^2.7^, respectively at enrollment. During a mean of 3.5 years, 692 patients developed composite outcomes (32.9%): 568 patients experienced renal events (27.0%), 130 patients experienced cardiovascular events (6.2%), and 80 patients died (3.8%).

We explored the baseline characteristics of the LVM quartile (Table 1). Age and the proportion of men increased with an increase in LVM. The proportion of currents smokers in the higher LVM quartile was greater. The proportion of patients with diabetic and hypertensive nephropathy increased, while that of patients with glomerulonephritis and other etiologies of CKD decreased, with the progression of the LVM quartile. Systolic and diastolic BP, BSA, BMI, fasting glucose, blood urea nitrogen, hsCRP, and UPCR increased, while eGFR, bilirubin, serum albumin, and total cholesterol decreased with an increase in LVM. Hemoglobin levels were not associated with LVM quartiles.

**Table 1:** Baseline characteristics of patients according to the status of left ventricular mass.

	Quartile of LVM				*P*-trend
	1 Quartile 59.0–128.0 g (n = 512)	2 Quartile 128.0–155.9 g (n = 536)	3 Quartile 155.9–186.4 g (n = 528)	4 Quartile 186.4–624.6 g (n = 525)	
Age (years)	49.4 ± 12.3	53.6 ± 12.3*	54.6 ± 11.5*	56.4 ± 11.8*†	<0.001
Male sex, n (%)	168, (32.8)	307, (57.3)*	374, (70.8)*†	433, (82.5)*†‡	<0.001
Current smoking, n (%)	45, (8.8)	82, (15.3)*	108, (20.5)*	103, (19.7)*	<0.001
Hypertension, n (%)	471, (92)	518, (96.6)*	522, (98.9)*	521, (99.2)*†	<0.001
Diabetes, n (%)	115, (22.7)	168, (31.6)*	212, (40.4)*†	249, (47.6)*†	<0.001
Cause of chronic kidney disease					
Diabetic nephropathy, n (%)	65, (12.7)	111, (20.7)*	162, (30.7)*†	183, (34.9)**†	<0.001
Hypertensive nephropathy, n (%)	73, (14.3)	111, (20.7)*	87, (16.5)	144, (27.5)*†‡	<0.001
Glomerulonephritis, n (%)	215, (42.0)	181, (33.8)*	167, (31.6)*	103, (19.7)*†‡	<0.001
Others (%)	159, (31.1)	133, (24.8)	112, (21.2)*	94, (17.9)*†	<0.001
Systolic BP (mm Hg)	122.1 ± 14.8	127.0 ± 14.9*	129.4 ± 15.8*	132.5 ± 17.4*†‡	<0.001
Diastolic BP (mm Hg)	75.7 ± 10.6	77.3 ± 10.3	76.9 ± 10.8	78.0 ± 12.3*	0.003
Weight (kg)	58.9 ± 10.0	65.6 ± 10.1*	68.9 ± 10.5*†	73.3 ± 11.7*†‡	<0.001
Height (cm)	160.5 ± 7.8	164.3 ± 8.3*	165.8 ± 8.0*	167.8 ± 7.9*†‡	<0.001
Body surface area (m^2^)	1.6 ± 0.2	1.7 ± 0.2*	1.8 ± 0.2*†	1.8 ± 0.2*†‡	<0.001
BMI (kg/m^2^)	22.8 ± 3.3	24.3 ± 3.1*	25.0 ± 3.1*†	26.0 ± 3.3*†‡	<0.001
LVM (g)	109.4 ± 13.7	141.5 ± 7.8*	169.8 ± 8.8*†	224.8 ± 42.9*†‡	<0.001
Fasting glucose (mmol/l)	5.8 ± 1.9	6.0 ± 1.8	6.3 ± 2.3*	6.4 ± 2.6*	<0.001
Blood urea nitrogen (mmol/l)	8.6 ± 4.7	9.3 ± 5.3	10.5 ± 5.5*†	11.7 ± 6.2*†‡	<0.001
Serum creatinine (μmol/l)	131.3 ± 75.6	147.6 ± 92.8	164.0 ± 94.3*	196.1 ± 125.3*†‡	<0.001
eGFR (ml/min/1.73m^2^)	61.8 ± 32.8	56.3 ± 30.9	51.3 ± 29.2*	44.3 ± 27.6*†‡	<0.001
Bilirubin (μmol/l)	11.7 ± 5.5	12.0 ± 5.4	11.3 ± 5.0	10.8 ± 5.0†	0.001
Serum albumin (g/l)	41.9 ± 4.1	42.4 ± 3.6	41.7 ± 4.5	41.1 ± 4.7†	<0.001
Total cholesterol (mmol/l)	4.6 ± 1.0	4.5 ± 1.0	4.5 ± 1.1	4.4 ± 1.0*	0.001
Hemoglobin (g/dl)	12.7 ± 1.8	13.1 ± 2*	12.9 ± 2.1	12.6 ± 2.1†	0.510
hsCRP (nmol/l)	3.8 (1.4–12.1)	5.7 (2.1–16.3)*	5.7 (2.7–14.3)*	8.2 (3.8–20.7)*†‡	<0.001
UPCR (g/g Cr)	0.4 (0.1–1.1)	0.4 (0.1–1.1)	0.5 (0.2–1.7)*†	0.8 (0.2–2.3)*†‡	<0.001

BP, blood pressure; BSA, body surface area; BMI, body mass index; LVM, left ventricular mass; eGFR, estimated glomerular filtration rate; hsCRP, high sensitivity C-reactive protein; UPCR, urine protein-to-creatinine ratio.

Values are expressed as mean ± standard deviation for normally distributed continuous variables, median (interquartile range) for non-normally distributed continuous variables, and percentage for categorical variables. *P*-trend was analyzed by linear-term of one-way ANOVA for normally distributed continuous variables, Jonckheere-Terpstra test for non-normally distributed continuous variables, and a linear-by-linear association for categorical variables.

*, †, and ‡ meant *P* < 0.01 when compared to 1Q-3Q of LVM group, respectively, by using Bonferroni post-hoc analysis of one-way ANOVA for normally distributed continuous variables, Mann-Whitney U test for non-normally distributed continuous variables, and chi-square test for categorical variables

We compared the echocardiographic parameters according to CKD staging (Table 2). LV chamber size (LVESD and LVEDD) and chamber thickness (IVST and PWT) increased, resulting in increased LVM, with the progression of CKD. Left atrial size (LAD) was also increased as CKD progressed. The prevalence of RWMA increased with the progression of CKD, although it was decreased in CKD stage 5. However, EF was not associated with the CKD stage.

**Table 2:** Trends of echocardiographic parameter according to CKD stages.

	CKD Stage (n = 2,101)						*P-*trend
	Stage 1(n = 344)	Stage 2 (n = 398)	Stage 3a (n = 344)	Stage 3b (n = 445)	Stage 4 (n = 444)	Stage 5 (n = 126)	
SBP (mmHg)	126 ± 14.3	126.4 ± 14.7	126.5 ± 15.8	126.8 ± 15.8	130.2 ± 17.1*†‡	135.7 ± 20.9*†‡¶§	<0.001
DBP (mmHg)	78.4 ± 10.8	77.6 ± 10.5	76.9 ± 10.5	75.9 ± 10.4	76.3 ± 12.2	77.4 ± 12.5	0.004
LVESD (cm)	3.0 ± 0.4	3.0 ± 0.4	3.0 ± 0.4	3.0 ± 0.4	3.1 ± 0.5†	3.1 ± 0.4	0.004
LVEDD (cm)	4.8 ± 0.4	4.8 ± 0.4	4.9 ± 0.4	4.9 ± 0.5	4.9 ± 0.5*†	4.9 ± 0.5	<0.001
IVST (cm)	0.9 ± 0.2	0.9 ± 0.2*	0.9 ± 0.2*	1.0 ± 0.2*†	1.0 ± 0.2*†	1.0 ± 0.2*†‡	<0.001
PWT (cm)	0.9 ± 0.1	0.9 ± 0.1	0.9 ± 0.1*	0.9 ± 0.1*†	1.0 ± 0.2*†‡	1.0 ± 0.2*†‡	<0.001
RWT	0.36 ± 0.06	0.38 ± 0.07	0.38 ± 0.06*	0.39 ± 0.07*	0.39 ± 0.08*	0.41 ± 0.09*†‡	<0.001
LVM (g)	145.7 ± 44.0	153.7 ± 40.7	158.6 ± 40.3*	165.4 ± 45.2*†	174.2 ± 57.6*†‡	180.0 ± 55.8*†‡	<0.001
LVMI-BSA (g/m^2^)	83.4 ± 20.5	87.4 ± 20.7	90.8 ± 20.4*	95.7 ± 22.8*†	101.3 ± 28.6*†‡¶	106.9 ± 29.0*†‡¶	<0.001
LVMI-H2.7 (g/m^2.7^)	37.1 ± 9.8	39.0 ± 10.0	41.0 ± 10.0*	43.5 ± 11.3*†	46.0 ± 13.4*†‡	49.0 ± 13.8*†‡¶	<0.001
EF (%)	64.0 ± 5.6	64.2 ± 6	63.9 ± 5.5	64.5 ± 5.8	63.8 ± 6.8	65.6 ± 6.2	0.288
RWMA, n (%)	4, (1.2)	8, (2.0)	10, (2.9)	18, (4.0)	22, (5.0)*	3, (2.4)	0.004
LAD (cm)	3.6 ± 0.5	3.7 ± 0.5	3.8 ± 0.6*	3.8 ± 0.6*	3.9 ± 0.6*†	3.9 ± 0.6*	<0.001

SBP, systolic blood pressure; DBP, diastolic blood pressure; CKD, chronic kidney disease; LVESD, left ventricular end systolic diameter; LVEDD, left ventricular end diastolic diameter; IVST, interventricular septum thickness; PWT, posterior wall thickness; RWT, relative wall thickness; LVM, left ventricular mass; LVMI, left ventricular mass index; BSA, body surface area; H2.7, height to the 2.7 power; EF, ejection fraction; RWMA, regional wall motion abnormality; LAD, left atrial diameter.

Values are expressed as mean ± standard deviation for continuous variables and percentage for categorical variables. *P*-trend was analyzed by linear-term of one-way ANOVA for continuous variables and a linear-by-linear association for categorical variables.

*, †, ‡, ¶, and § meant *P* < 0.01 when compared to CKD stage 1, 2, 3a, 3b, and 4 by using Bonferroni post-hoc analysis of one-way ANOVA for continuous variables and chi-square test for categorical variables.

**Fig 1:**
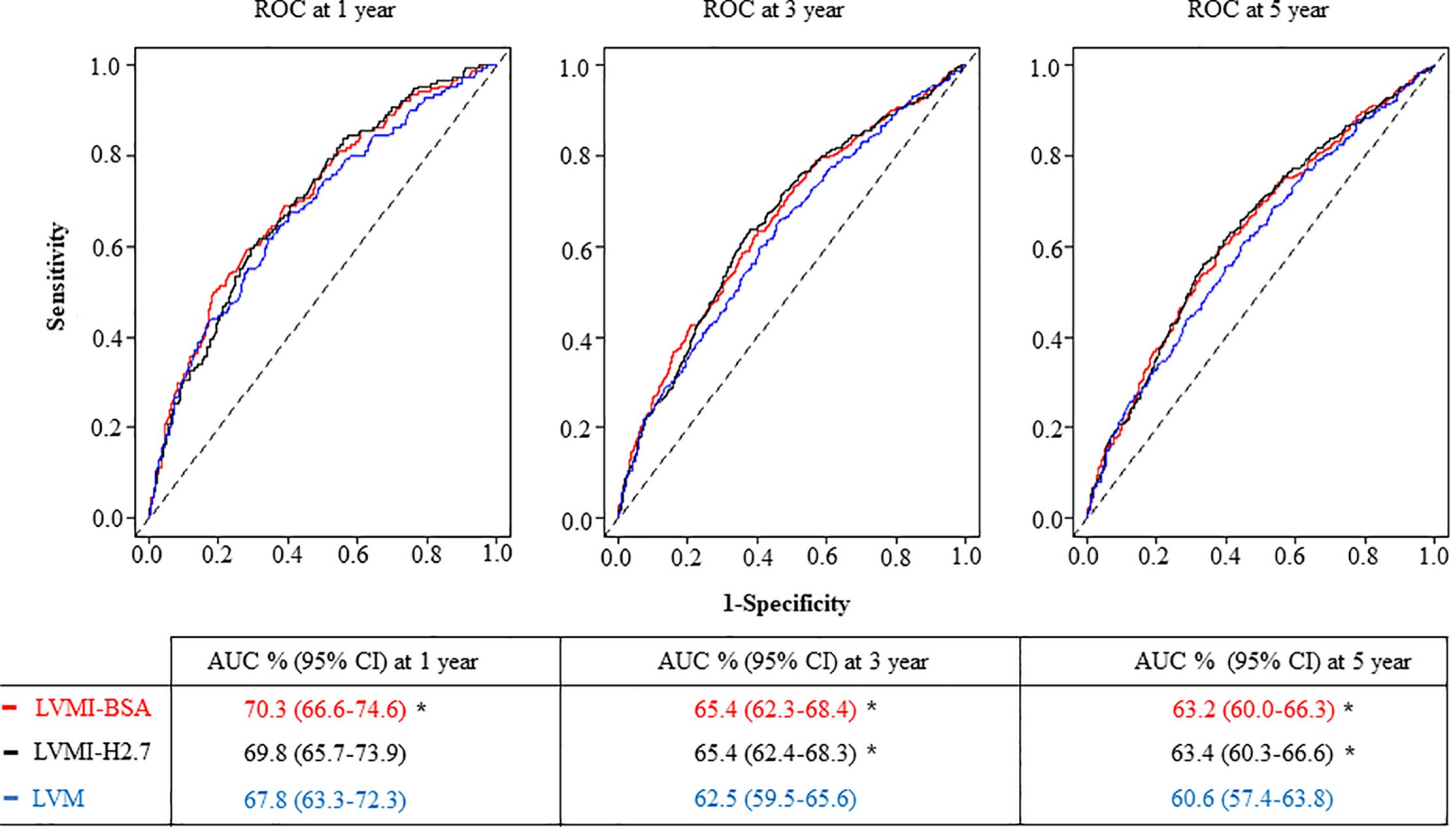
Time-dependent receiver operator characteristics curve of left ventricular mass and its indexations for composite outcome. AUC, area under the curve; CI, confidence interval; LVMI-BSA, left ventricular mass index by body surface area; LVMI-H2.7, left ventricular mass index by height to the 2.7 power; LVM, left ventricular mass; CV, cardiovascular. * meant *P* <0.05 when compared to LVM. The *P*-values of the comparison between AUCs of LVMI-BSA and LVMI-H2.7 were all above 0.05.

We analyzed the risk of LVM and its indexations for adverse clinical outcomes (Table 3). Increased LVM, LVMI-BSA, and LVMI-H2.7 were significantly associated with all clinical outcomes on univariate analysis. We performed multivariate analysis after adjusting for the effects of confounders and found that increased LVM and its indexations were independently associated with an increased risk of composite, renal, and cardiovascular outcomes (Table 3 and S1 Table). On the other hand, LVM and its indexations were not associated with all-cause mortality, according to multivariate analysis. We compared the AUCs with time-dependent ROC analysis, to identify the relative predictive ability of LVM and its indexations for composite outcome as the follow-up time increased (Fig 1). The AUCs of LVMI-BSA and LVMI-H2.7 for composite outcome were not different, and both were statistically greater than that of LVM, which had become more evident with the increase of follow-up time.

**Table 3:** Hazard ratios of left ventricular mass and its several indexations for adverse clinical outcomes.

	Univariate		Multivariate	
	HR (95% CI)	*P*	HR (95% CI)	*P*
**Composite outcome**				
LVM (g)	1.007 (1.005–1.008)	<0.001	1.004 (1.002–1.005)	<0.001
LVMI-BSA (g/m^2^)	1.019 (1.015–1.022)*	<0.001	1.011 (1.006–1.016)*	<0.001
LVMI-H2.7 (g/m^2.7^)	1.042 (1.033–1.051)*	<0.001	1.023 (1.012–1.035)*	<0.001
**Renal outcome**				
LVM (g)	1.009 (1.007–1.011)*	<0.001	1.006 (1.003–1.009)*	<0.001
LVMI-BSA (g/m^2^)	1.022 (1.017–1.026)*	<0.001	1.015 (1.009–1.020)*	<0.001
LVMI-H2.7 (g/m^2.7^)	1.049 (1.039–1.059)*	<0.001	1.032 (1.019–1.045)*	<0.001
**CV outcome**				
LVM (g)	1.006 (1.003–1.008)	<0.001	1.005 (1.000–1.009)	0.037
LVMI-BSA (g/m^2^)	1.014 (1.009–1.019)	<0.001	1.010 (1.002–1.017)	0.012
LVMI-H2.7 (g/m^2.7^)	1.031 (1.020–1.043)	<0.001	1.023 (1.006–1.040)	0.007
**All-cause mortality**				
LVM (g)	1.006 (1.002–1.009)	0.001	1.001 (0.995–1.006)	0.765
LVMI-BSA (g/m^2^)	1.014 (1.007–1.021)	<0.001	0.999 (0.990–1.009)	0.905
LVMI-H2.7 (g/m^2.7^)	1.026 (1.010–1.042)	0.001	0.996 (0.975–1.018)	0.710

LVM, left ventricular mass; LVMI, left ventricular mass index; BSA, body surface area; H, height; W, weight; CV, cardiovascular. HR and its CI were calculated using Cox proportional hazard regression analysis. In multivariate analysis, covariates were age, sex, current smoking, causes of chronic kidney disease, systolic blood pressure ≥ 127 mmHg, diastolic blood pressure ≥ 77 mmHg, blood urea nitrogen≥ 8.6 mmol/l, estimated glomerular filtration rate ≥ 46.3 ml/min/1.73m^2^, bilirubin ≥ 10.3 μmol/l, albumin ≥ 42 g/l, cholesterol ≥ 4.4 mmol/l, hemoglobin ≥ 12.8 g/dl, body mass index, fasting glucose, urine protein creatinine ratio, and high sensitive C-reactive protein.

* meant results using time-dependent Cox hazard regression analysis.

**Fig 2:**
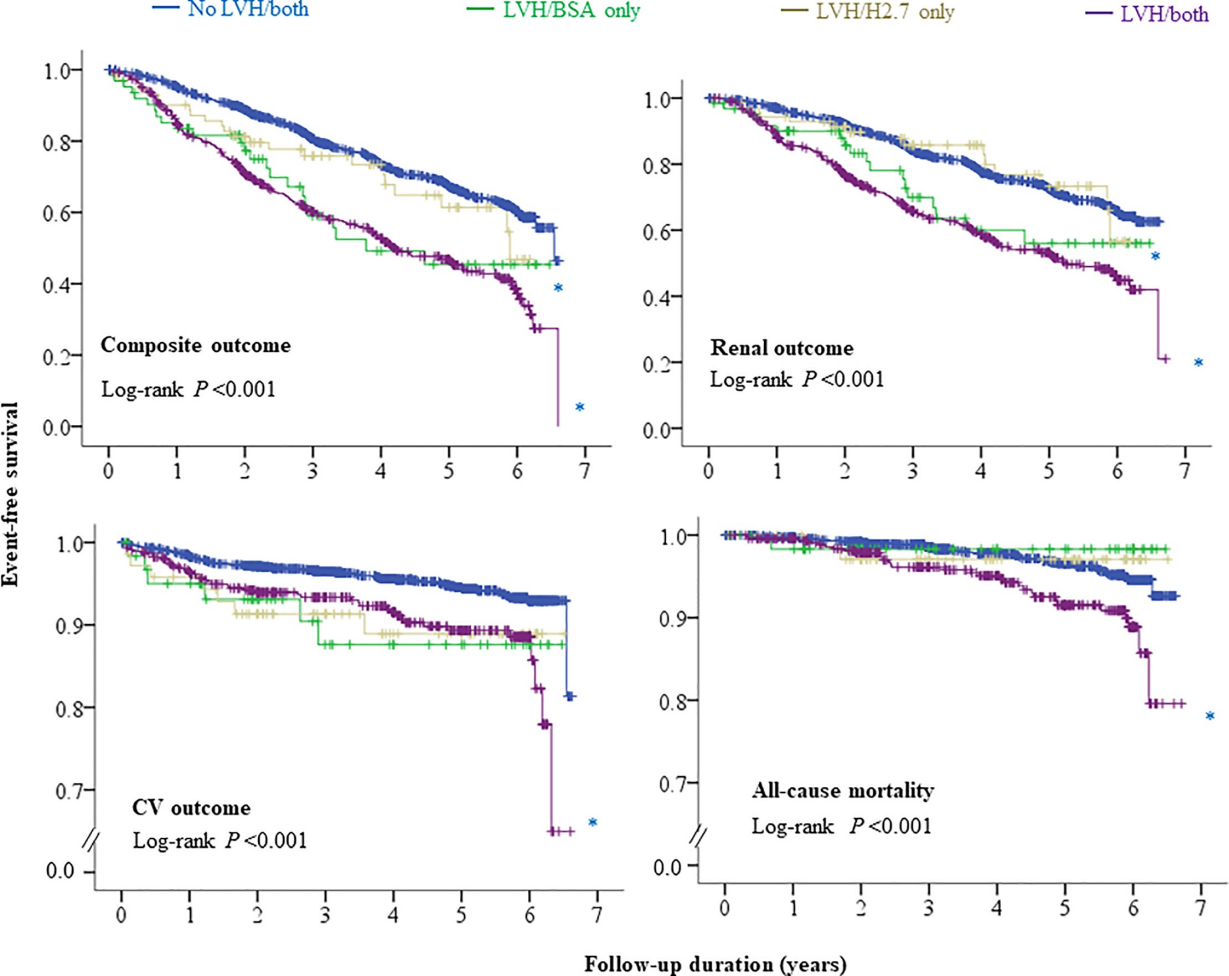
Kaplan Meier survival curve of left ventricular hypertrophy (LVH) groups defined by left ventricular mass index by body surface area (BSA) or height to the 2.7 power (H2.7) for adverse clinical outcomes. *, †, and ‡ meant *P* < 0.01 when compared to No LVH/both, LVH/BSA only, and LVH/H2.7 only groups, respectively, by using log-rank test.

We compared the risk of the four LVH groups for adverse clinical outcomes. We confirmed that the LVH/BSA-only and LVH/both groups showed independent risks for composite and renal outcomes, while no risk was observed in the LVH/H2.7-only group, when compared to the no LVH/both group, according to multivariate Cox proportional hazard regression analysis (Table 4 and S2 Table). Kaplan-Meier’s survival curve analysis (Fig 2) revealed that the LVH/BSA-only and LVH/both groups showed significantly lower event-free survival for composite and renal outcomes, while the LVH/H2.7 only group showed similar event-free survival for composite and renal outcomes, compared to no LVH/both group. The LVH/both group showed significantly lower event-free survival for cardiovascular outcomes and all-cause mortality, compared to the no LVH/both group. However, this was not confirmed with multivariate analysis (Table 4 and S2 Table).

**Table 4:** Hazard ratio of left ventricular hypertrophy (LVH) groups defined by left ventricular mass index by body surface area or height to the 2.7 power for adverse clinical outcomes.

	Composite outcome		Renal outcome		CV outcome		All-cause mortality	
LVH groups	HR (95% CI)	*P*	HR (95% CI)	*P*	HR (95% CI)	*P*	HR (95% CI)	*P*
No LVH/Both (n = 1,372)	Ref.		Ref.		Ref.		Ref.	
LVH/BSA only (n = 56)	1.908 (1.233–2.953)	0.004	1.820 (1.109–2.986)	0.018	2.484 (0.978–6.307)	0.056	0.519 (0.070–3.866)	0.522
LVH/H2.7 only (n = 64)	1.079 (0.680–1.712)	0.747	0.835 (0.478–1.457)	0.525	1.696 (0.694–4.145)	0.247	0.766 (0.171–3.424)	0.727
LVH/both (n = 410)	1.352 (1.123–1.626)	0.001	1.414 (1.155–1.730)	0.001	1.299 (0.839–2.011)	0.240	1.133 (0.676–1.900)	0.635

LVH, left ventricular hypertrophy; BSA, body surface area; H2.7, height to the 2.7 power; CV, cardiovascular; Ref, reference; HR, hazard ratio; CI, confidence interval. HR and its CI were analyzed using multivariate Cox proportional hazard regression analysis entering into age, sex, current smoking, causes of chronic kidney disease, systolic blood pressure ≥ 127 mmHg, diastolic blood pressure ≥ 77 mmHg, blood urea nitrogen≥ 8.6 mmol/l, estimated glomerular filtration rate ≥ 46.3 ml/min/1.73m^2^, bilirubin ≥ 10.3 μmol/l, albumin ≥ 42 g/l, cholesterol ≥ 4.4 mmol/l, hemoglobin ≥ 12.8 g/dl, body mass index, fasting glucose, urine protein creatinine ratio, and high sensitive C-reactive protein as covariates.

## Discussion

Increased LVM can predict cardiovascular events and mortality in patients with [23–27] or without CKD [5, 28–34] LVM can increase physiologically in individuals with a large body size (11). Therefore, the need for indexing LVM has been suggested for better calculation of LVM by minimizing the effect of body size [10, 11, 13, 16, 35–38] However, whether calculation of LVM using several indexations results in improvement in predictions of adverse clinical outcomes needs investigation. This study compared the AUCs of LVM and its indexations for several clinical outcomes and found that LVM indexing with BSA or height^2.7^ significantly improved the predictive power for renal and cardiovascular events. We also analyzed the association between LVMs indexed with BSA and height^2.7^ and clinical outcomes. Both LVMI-BSA and LVMI-H2.7 independently predicted the development of renal and cardiovascular events. However, LVM and its indexations were not associated with all-cause mortality, which may be attributed to the overall low mortality in this population [39].

Several indexing methods exist, including height^2.7 13^, BSA^1.5^, and fat-free mass [11, 12, 35], among which, BSA and height^2.7^ indexations are the most studied [10, 11, 13, 16, 35–38]. Although it is obvious that the prevalence of LVH, as defined by LVMI-BSA and LVMI-H2.7 can vary [36–38] the impact of different classifications of LVH using different LVMIs on adverse clinical outcomes has been studied scarcely, with inconclusive results [10, 12, 16]. Moreover, the impact of LVM and its indexations on renal events has been rarely studied [40]. In this study, both LVMI-BSA and LVMI-H2.7 were independently associated with renal and cardiovascular events, and composite outcome. The AUCs of time-dependent ROC of LVMI-BSA and LVMI-H2.7 for composite outcome were comparable, which was largely attributed to the relationship with renal and cardiovascular events (S3 Table). Net reclassification improvement of LVMI-H2.7 over LVMI-BSA for composite outcome was not statistically significant (S4 Table). In the analysis for the diagnostic performance of LVMI-BSA and LVMI-H2.7 for LVH using the highest sex-specific quartiles for the respective LVMIs, the LVH/both group was undoubtedly independently associated with an increased risk of composite and renal events. Although the LVH/H2.7-only group was not associated with composite and renal events, the LVH/BSA only group showed a significantly high risk for composite and renal events, despite having very few patients. Therefore, we assumed that BSA may be as useful as height^2.7^ in indexing LVM in pre-dialysis CKD patients.

Our results were discordant with the study by Zoccali et al. (16). They analyzed 254 patients undergoing dialysis and reported that height^2.7^ provides better indexation of LVM than BSA for predicting overall and cardiovascular mortality and cardiovascular events. We assumed that difference in the volume status, based on the dialysis status of patients with CKD may be responsible for different results. Unlike pre-dialysis patients with CKD [41], dialysis patients tend to have a greater volume overload [15] and dialysis procedure affected much on volume status and echocardiographic measures [42]. In subgroup analysis according to CKD stages (S5 Table), LVMI-BSA was significantly associated with composite outcome in groups with stage 3a-b and stage 4–5, while LVMI-H.27 was associated with composite outcome only in group with stage 4–5. Although effect modification of CKD stages on the relationship between LVM indexations and composite outcome was marginal, which might be because subjects with CKD stage 1–2 had very low rates of adverse events, it is obvious that volume is more likely to be overloaded with the progression of CKD stage [43]. Therefore, the evident relationship between LVMI-H2.7 and composite outcome only in advanced CKD may be in line with the results from Zoccali et al. and the poorer performance of LVMI-BSA in the study by Zoccali et al. [16] may be attributed to the high volume status in dialysis patients, which was in line with the poorer performance of BSA in populations with obesity [35, 36].

This study had several strengths. First, this was the largest study to examine the clinical significance of LVM and its indexation on adverse clinical outcomes in pre-dialysis patients with CKD. The study was the first to demonstrate the association between LVM and its indexations and renal events. Second, missing rates of major variables were low. Third, the study results support the current ASE guideline, which uses BSA as a major indexation [1, 14]. The LVH is important cardiovascular risk factor in CKD patients and many studies have been done. The validation which LVM index is better could help researchers to use unified LVMI and facilitate to compare the studies. According to our results, we suggest to use the LVMI-BSA in the pre-dialysis CKD patients. The study also had several limitations. First, the cardiovascular events and all-cause mortality were too low, despite a moderate follow-up duration [39]. Therefore, the null association of LVM and its indexations with all-cause mortality needs to be re-evaluated, when a considerable number of events are developed. Second, we did not evaluate markers for the volume status. Although we had echocardiographic measures, cardiac geometry analysis showed that both eccentric and concentric stresses were increased with the progression of CKD stages (S6 Table). Therefore, the effect of volume overload in pre-dialysis patients with CKD could not be presented in this study. Third, the common ethnicity of the study’s participants limits the generalizability of the results.

In conclusion, LVM indexing improved the predictive ability of future adverse outcomes. BSA may be as useful as height^2.7^ in indexing LVM in pre-dialysis patients with CKD for predicting future adverse outcomes. Subsequent studies are needed to confirm our results. 

## Supporting information

S1 Table Serial adjustment of left ventricular mass and its several indexations for adverse clinical outcomes. (DOCX)

S2 Table Hazard ratio of left ventricular hypertrophy (LVH) groups defined by left ventricular mass index by body surface area or height to the 2.7 power for adverse clinical outcomes. (DOCX)

S3 Table Time-dependent ROC analysis between LVM and its indexations for renal and cardiovascular events and all-cause death. (DOCX)

S4 Table Net reclassification improvement of LVMI-H2.7 compared to LVMI-BSA for composite outcome. (DOCX)

S5 Table Hazard ratios of left ventricular mass and its several indexations for composite outcome. (DOCX)

S6 Table Cardiac geometry and stages of chronic kidney disease. (DOCX)
