# Supplementary material for: Indexation of left ventricular mass to predict adverse clinical outcomes in pre-dialysis patients with chronic kidney disease: KoreaN cohort study of the outcome in patients with chronic kidney disease
Source: PLoS One. 2020 May 19;15(5):e0233310. doi: 10.1371/journal.pone.0233310 (PMC7236996; doi:10.1371/journal.pone.0233310)
Supplement: S1 Table — (DOCX) [file pone.0233310.s001.docx]

Table S1. Serial adjustment of left ventricular mass and its several indexations for adverse clinical outcomes

|  | **Model 1** | | **Model 2** | |
| --- | --- | --- | --- | --- |
|  | HR (95% CI) | *P* | HR (95% CI) | *P* |
| **Composite outcome** |  |  |  |  |
| LVM (g) | 1.007 (1.005-1.008) | <0.001 | 1.007 (1.005-1.008) | <0.001 |
| LVMI-BSA (g/m^2^)* | 1.019 (1.015-1.023) | <0.001 | 1.018 (1.014-1.022) | <0.001 |
| LVMI-H2.7 (g/m^2.7^)* | 1.041 (1.031-1.051) | <0.001 | 1.040 (1.030-1.050) | <0.001 |
| **Renal outcome** |  |  |  |  |
| LVM (g)* | 1.010 (1.008-1.012) | <0.001 | 1.010 (1.007-1.012) | <0.001 |
| LVMI-BSA (g/m^2^)* | 1.022 (1.018-1.026) | <0.001 | 1.021 (1.017-1.026) | <0.001 |
| LVMI-H2.7 (g/m^2.7^)* | 1.049 (1.039-1.060) | <0.001 | 1.048 (1.037-1.059) | <0.001 |
| **CV outcome** |  |  |  |  |
| LVM (g) | 1.005 (1.001-1.008) | 0.010 | 1.004 (1.000-1.008) | 0.043 |
| LVMI-BSA (g/m^2^) | 1.010 (1.004-1.017) | 0.002 | 1.009 (1.002-1.016) | 0.011 |
| LVMI-H2.7 (g/m^2.7^) | 1.022 (1.008-1.036) | 0.002 | 1.021 (1.006-1.036) | 0.006 |
| **All-cause mortality** |  |  |  |  |
| LVM (g) | 1.004 (0.999-1.008) | 0.124 | 1.005 (1.000-1.010) | 0.048 |
| LVMI-BSA (g/m^2^) | 1.008 (0.999-1.017) | 0.080 | 1.008 (0.999-1.017) | 0.086 |
| LVMI-H2.7 (g/m^2.7^) | 1.009 (0.991-1.028) | 0.321 | 1.015 (0.995-1.035) | 0.135 |

LVM, left ventricular mass; LVMI, left ventricular mass index; BSA, body surface area; H, height; W, weight; CV, cardiovascular. HR and its CI were calculated using Cox proportional hazard regression analysis. In model 1, covariates were age and sex. In model 2, current smoking, causes of chronic kidney disease, systolic blood pressure ≥ 127 mmHg, diastolic blood pressure ≥ 77 mmHg, cholesterol ≥ 4.4 mmol/l, body mass index and fasting glucose were added as covariates in addition to model 1.* meant results using time-dependent Cox hazard regression analysis.
