# Supplementary material for: Indexation of left ventricular mass to predict adverse clinical outcomes in pre-dialysis patients with chronic kidney disease: KoreaN cohort study of the outcome in patients with chronic kidney disease
Source: PLoS One. 2020 May 19;15(5):e0233310. doi: 10.1371/journal.pone.0233310 (PMC7236996; doi:10.1371/journal.pone.0233310)
Supplement: S2 Table — (DOCX) [file pone.0233310.s002.docx]

Table S2. Hazard ratio of left ventricular hypertrophy (LVH) groups defined by left ventricular mass index by body surface area or height to the 2.7 power for adverse clinical outcomes.

|  | **Composite outcome** | | **Renal outcome** | | **CV outcome** | | **All-cause mortality** | |
| --- | --- | --- | --- | --- | --- | --- | --- | --- |
|  | HR (95% CI) | *P* | HR (95% CI) | *P* | HR (95% CI) | *P* | HR (95% CI) | *P* |
| **Model 1** |  |  |  |  |  |  |  |  |
| No LVH/Both (n = 1,372) | Ref. |  | Ref. |  | Ref. |  | Ref. |  |
| LVH/BSA only (n = 56) | 2.032 (1.346-3.068) | 0.001 | 1.667 (1.038-2.678) | 0.035 | 2.845 (1.235-6.554) | 0.014 | 0.663 (0.091-4.822) | 0.685 |
| LVH/H2.7 only (n = 64) | 1.210 (0.793-1.845) | 0.377 | 0.960 (0.571-1.614) | 0.877 | 1.595 (0.730-3.483) | 0.242 | 0.656 (0.158-2.719) | 0.561 |
| LVH/both (n = 410) | 1.974 (1.666-2.339) | <0.001 | 2.130 (1.769-2.566) | <0.001 | 1.443 (0.981-2.123) | 0.062 | 1.504 (0.943-2.397) | 0.087 |
| **Model 2** |  |  |  |  |  |  |  |  |
| No LVH/Both (n = 1,372) | Ref. |  | Ref. |  | Ref. |  | Ref. |  |
| LVH/BSA only (n = 56) | 1.997 (1.320-3.023) | 0.001 | 1.761 (1.093-2.837) | 0.020 | 2.545 (1.091-5.935) | 0.031 | 0.537 (0.073-3.946) | 0.541 |
| LVH/H2.7 only (n = 64) | 1.151 (0.737-1.798) | 0.537 | 0.859 (0.495-1.490) | 0.588 | 1.616 (0.711-3.670) | 0.252 | 0.949 (0.220-4.089) | 0.943 |
| LVH/both (n = 410) | 1.783 (1.499-2.120) | <0.001 | 1.907 (1.577-2.307) | <0.001 | 1.349 (0.902-2.016) | 0.145 | 1.659 (1.025-2.684) | 0.039 |

LVH, left ventricular hypertrophy; BSA, body surface area; H2.7, height to the 2.7 power; CV, cardiovascular; Ref, reference; HR, hazard ratio; CI, confidence interval. HR and its CI were analyzed using multivariate Cox proportional hazard regression analysis. In model 1, covariates were age and sex. In model 2, current smoking, causes of chronic kidney disease, systolic blood pressure ≥ 127 mmHg, diastolic blood pressure ≥ 77 mmHg, cholesterol ≥ 4.4 mmol/l, body mass index and fasting glucose were added as covariates in addition to model 1.
