# Supplementary material for: Indexation of left ventricular mass to predict adverse clinical outcomes in pre-dialysis patients with chronic kidney disease: KoreaN cohort study of the outcome in patients with chronic kidney disease
Source: PLoS One. 2020 May 19;15(5):e0233310. doi: 10.1371/journal.pone.0233310 (PMC7236996; doi:10.1371/journal.pone.0233310)
Supplement: S3 Table — (DOCX) [file pone.0233310.s003.docx]

Table S3. Time-dependent ROC analysis between LVM and its indexations for renal and cardiovascular events and all-cause death.

|  |  | ROC at 1 year | | | ROC at 3 year | | | ROC at 5 year | | |
| --- | --- | --- | --- | --- | --- | --- | --- | --- | --- | --- |
|  |  | AUC (95% CI) | *P* | *P* | AUC (95% CI) | *P* | *P* | AUC (95% CI) | *P* | *P* |
| **Renal events** | LVMI-BSA | 73.5 (68.8-78.3) | **<0.001** | 0.065 | 65.6 (62.4-68.9) | **<0.001** | 0.934 | 62.6 (59.3-65.9) | **<0.001** | 0.875 |
|  | LVMI-H2.7 | 72.0 (67.2-76.8) | 0.132 | Ref. | 65.6 (62.3-68.8) | **<0.001** | Ref. | 62.7 (59.4-66.0) | **0.007** | Ref. |
|  | LVM | 69.8 (64.7-75.0) | Ref. |  | 62.3 (59.0-65.6) | Ref. |  | 60.2 (56.8-63.5) | Ref. |  |
| **CV events** | LVMI-BSA | 63.1 (54.8-71.4) | 0.771 | 0.073 | 61.7 (55.8-67.7) | 0.102 | 0.555 | 63.0 (57.4-68.6) | **0.011** | 0.187 |
|  | LVMI-H2.7 | 65.5 (58.1-72.8) | 0.142 | Ref. | 62.4 (56.8-67.9) | 0.149 | Ref. | 64.1 (58.6-69.5) | **0.007** | Ref. |
|  | LVM | 62.8 (54.4-71.1) | Ref. |  | 60.3 (54.3-66.2) | Ref. |  | 60.3 (54.6-65.9) | Ref. |  |
| **All-cause death** | LVMI-BSA | 70.3(48.8-91.7) | 0.305 | **0.001** | 64.0 (54.4-73.6) | 0.306 | 0.870 | 61.9 (55.3-70.3) | **0.010** | 0.302 |
|  | LVMI-H2.7 | 62.7 (43.5-81.9) | **0.001** | Ref. | 63.8 (54.5-73.0) | 0.530 | Ref. | 61.9 (54.4-69.4) | 0.190 | Ref. |
|  | LVM | 73.0 (51.0-95.0) | Ref. |  | 62.4 (52.9-72.0) | Ref. |  | 59.6 (52.2-67.1) | Ref. |  |

ROC, receiver operating characteristic curve; LVM, left ventricular mass; LVMI, left ventricular mass index; BSA, body surface area; H.27 height powered to 2.7; AUC, area under the curve; CI, confidence interval.
