# Supplementary material for: Indexation of left ventricular mass to predict adverse clinical outcomes in pre-dialysis patients with chronic kidney disease: KoreaN cohort study of the outcome in patients with chronic kidney disease
Source: PLoS One. 2020 May 19;15(5):e0233310. doi: 10.1371/journal.pone.0233310 (PMC7236996; doi:10.1371/journal.pone.0233310)
Supplement: S4 Table — (DOCX) [file pone.0233310.s004.docx]

Table S4. Net reclassification improvement of LVMI-H2.7 compared to LVMI-BSA for composite outcome

|  | Categorical NRI (95% CI) | *P* | Continuous NRI (95% CI) | *P* |
| --- | --- | --- | --- | --- |
| Univariate | 0.005 (-0.025 to 0.035) | 0.751 | -0.013 (-0.109 to 0.082) | 0.785 |
| Multivariate | 0.005 (-0.002 to 0.011) | 0.159 | 0.024 (-0.072 to 0.119) | 0.628 |

LVMI, left ventricular mass index; BSA, body surface area; H2.7, height to the 2.7 power; NRI, net reclassification improvement. For multivariate analysis, we built up a basic model entering into age, sex, current smoking, causes of chronic kidney disease, systolic and diastolic blood pressure, blood urea nitrogen, estimated glomerular filtration rate, bilirubin, albumin, cholesterol, hemoglobin, body mass index, fasting glucose, urine protein creatinine ratio, and high sensitive C-reactive protein as covariates. Subsequently, two models adding LVMI-BSA and LVMI-H2.7 were generated and NRI analysis was done.
