# Supplementary material for: Indexation of left ventricular mass to predict adverse clinical outcomes in pre-dialysis patients with chronic kidney disease: KoreaN cohort study of the outcome in patients with chronic kidney disease
Source: PLoS One. 2020 May 19;15(5):e0233310. doi: 10.1371/journal.pone.0233310 (PMC7236996; doi:10.1371/journal.pone.0233310)
Supplement: S5 Table — (DOCX) [file pone.0233310.s005.docx]

Table S5. Hazard ratios of left ventricular mass and its several indexations for composite outcome

| Exposure | Subgroups: CKD stages | Adjusted HR (95% CI) | *P* | *P for interaction* |
| --- | --- | --- | --- | --- |
| LVMI-BSA (g/m^2^) | Stage 1-2 (n = 742) | 1.009 (0.989-1.029) | 0.384 | 0.096 |
|  | **Stage 3a-b (n= 479)** | **1.031 (1.005-1.058)** | **0.021** |  |
|  | **Stage 4-5 (n = 880)** | **1.009 (1.003-1.014)** | **0.003** |  |
| LVMI-H2.7 (g/m^2.7^) | Stage 1-2 (n = 742) | 1.026 (0.985-1.069) | 0.223 | 0.080 |
|  | Stage 3a-b (n= 479) | 1.049 (0.995-1.106) | 0.078 |  |
|  | **Stage 4-5 (n = 880)** | **1.019 (1.006-1.031)** | **0.004** |  |

LVMI, left ventricular mass index; BSA, body surface area; H2.7, height to the 2.7 power; HR, hazard ratio; CI, confidence interval. HR and its CI were calculated using time dependent Cox proportional hazard regression analysis. In multivariate analysis, covariates were age, sex, current smoking, causes of chronic kidney disease, systolic blood pressure ≥ 127 mmHg, diastolic blood pressure ≥ 77 mmHg, blood urea nitrogen≥ 8.6 mmol/l, estimated glomerular filtration rate ≥ 46.3 ml/min/1.73m^2^, bilirubin ≥ 10.3 μmol/l, albumin ≥ 42 g/l, cholesterol ≥ 4.4 mmol/l, hemoglobin ≥ 12.8 g/dl, body mass index, fasting glucose, urine protein creatinine ratio, and high sensitive C-reactive protein.
