# Supplementary material for: Indexation of left ventricular mass to predict adverse clinical outcomes in pre-dialysis patients with chronic kidney disease: KoreaN cohort study of the outcome in patients with chronic kidney disease
Source: PLoS One. 2020 May 19;15(5):e0233310. doi: 10.1371/journal.pone.0233310 (PMC7236996; doi:10.1371/journal.pone.0233310)
Supplement: S6 Table — (DOCX) [file pone.0233310.s006.docx]

Table S6. Cardiac geometry and stages of chronic kidney disease

|  | CKD Stage (n =2,101) | | | | | | *P-*trend |
| --- | --- | --- | --- | --- | --- | --- | --- |
|  | Stage 1(n = 344) | Stage 2 (n = 398) | Stage 3a (n = 344) | Stage 3b (n =445 ) | Stage 4 (n =444 ) | Stage 5 (n =126 ) |  |
| Concentric LVH, n (%) | 18, (5.2) | 31, (7.8) | 35, (10.2) | 56, (12.6)* | 83, (18.7)*† | 32, (25.4)*†‡¶ | <0.001 |
| Eccentric LVH, n (%) | 24, (7) | 38, (9.5) | 33, (9.6) | 57, (12.8)* | 87, (19.6)*† | 22, (17.5)* | <0.001 |
| Concentric Remodelling, n (%) | 35, (10.2) | 58, (14.6) | 59, (17.2)* | 79, (17.8)* | 58, (13.1) | 15, (11.9) | 0.204 |

LVH, left ventricular hypertrophy. Concentric LVH was defined as LVMI-BSA > 115 g/m^2^ (men) or >95 g/m^2^ and relative wall thickness (RWT) > 0.42, eccentric LVH was defined as LVMI-BSA > 115 g/m^2^ (men) or >95 g/m^2^ and RWT ≤0.42, and concentric remodeling was defined as LVMI-BSA ≤ 115 g/m^2^ (men) or ≤95 g/m^2^ and RWT > 0.42, following the recent recommendation from a Report from the European Association of Cardiovascular Imaging (EACVI) and the American Society of Echocardiography (ASE). *, †, ‡, ¶, and § meant *P* < 0.01 when compared to CKD stage 1, 2, 3a, 3b, and 4 by using Bonferroni post-hoc analysis of one-way ANOVA for continuous variables and chi-square test for categorical variables.
